# A systematic review and meta-analysis of salmonellosis in poultry farms in Ethiopia: prevalence, risk factors, and antimicrobial resistance

**DOI:** 10.3389/fvets.2025.1538963

**Published:** 2025-05-28

**Authors:** Eyoel Basazinew, Haileyesus Dejene, Gashaw Getaneh Dagnaw, Asres Zegeye Lakew, Abebe Tesfaye Gessese

**Affiliations:** ^1^Department of Biomedical Sciences, College of Veterinary Medicine and Animal Sciences, University of Gondar, Gondar, Ethiopia; ^2^Department of Veterinary Epidemiology and Public Health, College of Veterinary Medicine and Animal Sciences, University of Gondar, Gondar, Ethiopia; ^3^Sirinka Agricultural Research Centre, Amhara Agricultural Research Institute, Bahir Dar, Ethiopia

**Keywords:** Ethiopia, meta-analysis, pooled prevalence, pooled resistance, poultry, Salmonella

## Abstract

Salmonellosis has a significant impact on the chicken production industry and is becoming a serious threat to public health. However, there is no systematic and inclusive report on the prevalence, associated risk factors, and antimicrobial resistance of chicken salmonellosis in Ethiopia. Therefore, the objective of this study was to estimate the pooled prevalence, identify possible risk factors, and assess antimicrobial resistance of *Salmonella* in poultry farms across Ethiopia. Studies were identified from databases such as Medline/PubMed, ScienceDirect/Scopus, Google Scholar, Web of Science, and Science Pub. The overall literature review and quantitative synthesis were conducted following the PRISMA guidelines. Overall, data extraction was conducted using Microsoft Excel, and statistical analysis was performed using R software. A total of 12 articles, published between August 2017 and October 2024, were included in the final quantitative synthesis. A random-effects meta-regression model was employed to estimate the pooled prevalence. The overall pooled prevalence of poultry salmonellosis was 12.46% (95% CI: 8.44, 16.48), with high heterogeneity (I^2^ = 97%, *τ*^2^ = 0.0041, *p* < 0.01). The subgroup meta-analysis of the study area showed that the prevalence proportion was higher in western Ethiopia, at 23.18% (95% CI: 8.96–37.39%). Based on the purpose of production, the highest pooled prevalence was observed in broilers at 28.23% (95% CI: 19.97–36.49%), while the highest prevalence based on age was in poultry under 6 months, at 14.45% (95% CI: 8.92–19.99%). Additionally, higher prevalence proportions were observed in local breeds and the Cobb 500 variety, with prevalence rates of 39.78% (95% CI: 19.50–60.06%) and 45.26% (95% CI: 23.44–67.08%), respectively. The highest pooled resistance levels for antimicrobials were observed against tetracycline (75%) (95% CI: 70–79%) and oxytetracycline (64%) (95% CI: 56–71%), while the lowest pooled resistance levels were against cefotaxime (3%) (95% CI: 0–7%) and gentamycin (6%) (95% CI: 4–9%). The results of the publication bias analysis showed the presence of asymmetry in the slope distribution, with no statistical difference. In conclusion, poultry salmonellosis is highly prevalent in Ethiopia. So, it is crucial to increase biosecurity and implement prevention and control methods to safeguard the health of poultry and humans in Ethiopia.

## 1 Introduction

In a developing country like Ethiopia, livestock is an integral part of agriculture, accounting for about 20% of the total GDP and 45% of the total value of agricultural production. It supports the livelihoods of a large proportion of the population (45), with the poultry sub-sector being particularly promising (1). Poultry Production plays a key role in reducing malnutrition and poverty, and in promoting economic growth among resource-poor households. It also contributes to the country’s economic development (46). Despite Ethiopians having one of the lowest poultry and poultry product consumption rates globally, the eggs and meat produced are still insufficient to meet the growing demand in metropolitan areas (54). The government’s 10-year perspective plan aims to significantly increase poultry meat production by 120.83% and boost egg production by 94.31% (44). These factors have collectively contributed to a rise in what are known as “production diseases” within poultry systems. Among these, the most important public health issue is salmonellosis (2).

Salmonellosis is caused by the rod-shaped (bacillus) gram-negative *Salmonella* bacteria of the family Enterobacteriaceae, a leading cause of enteric bacterial diseases in avian species (3). *Salmonella enterica* has six known subspecies, that are enterica, salamae, arizonae, diarizonae, houtenae, and indica (4). The majority of the *Salmonella* species that are significant animal diseases are found in *Salmonella enterica* subspecies enterica (5).

Salmonellosis is one of the most significant poultry diseases due to its substantial economic impact, global distribution, and the challenges associated with its control. Salmonellosis reduces chicken production in Ethiopia, causing 20–50% mortality from day-old to adulthood (6).

In poultry farming, multiple factors contribute to the occurrence of *Salmonella* on farms, including large flock sizes, poor management practices, sourcing chickens from various multiplication centers, using floor housing systems, and rearing layer chickens (7). Effective vaccination programs and biosecurity measures are important for preventing further transmission. Every effort should be made to eradicate *Salmonella*, with treatment as the last option (37). Various sulfonamides, followed by nitrofurans and other antibiotics such as furaltadone, furazolidone, chloramphenicol, neomycin, apramycin, gentamicin, and chlortetracycline, are effective in reducing mortality from *Salmonella*. However, this treatment option has a downside effect such as antimicrobial resistance (8).

Over the past few decades, there has been an alarming increase in antibiotic-resistant bacteria due to poor management of poultry farm and inappropriate use of antibiotics. According to Abdi et al. (9), 45 *Salmonella* isolates with various serotype were subjected to antimicrobial susceptibility testing, and all 45 isolates (100%) were found to be resistant to kanamycin and sulfamethoxazole-trimethoprim. Furthermore, 31 isolates were susceptible to ciprofloxacin and gentamicin. These indicates that resistance genes and bacteria have emerged and are being maintained in the chicken environment due to the ongoing use of antimicrobial medications in poultry production (10).

Despite various reports highlighting the prevalence and antimicrobial challenges of salmonellosis in poultry farms in Ethiopia, comprehensive data on its prevalence, associated factors, and antimicrobial resistance remain lacking. Therefore, this systematic review and meta-analysis aimed to estimate the pooled prevalence of salmonellosis in chickens, assess the pooled antimicrobial resistance of the isolates, and identify the contributing risk factors in chickens in Ethiopia.

## 2 Methodology

### 2.1 Study protocols

This systematic review and meta-analysis were conducted from August 2024 to October 2024, following the Preferred Reporting Items for Systematic Reviews and Meta-Analyses (PRISMA) guidelines (11) (Supplementary File 1). The outcomes of interest were the pooled prevalence of poultry salmonellosis in Ethiopia, estimates of the pooled prevalence among different subgroups, and drug resistance.

### 2.2 Literature search strategy and source of information

Published articles were used as sources of information for this study. The articles included in this systematic review and meta-analysis were searched from various electronic databases, including Medline/PubMed, ScienceDirect/Scopus, Google Scholar, Web of Science, and SciencePub, until September 10, 2024. Article searches were conducted using medical subject heading (MeSH) terms combined with Boolean operators (AND and OR). The following search terms were used: “Salmonellosis” OR “*Salmonella*” AND “seroprevalence” OR “prevalence” OR “epidemiology” AND “poultry” OR “chick” OR “chicken” OR “broilers” OR “laying hen” AND “risk factors” OR “potential factor” AND “antimicrobial resistance” OR “antibiotic resistance” OR “antimicrobial susceptibility” OR “drug susceptibility” AND “Ethiopia.”

### 2.3 Inclusion and exclusion criteria

The objectives of the study served as the basis for defining the inclusion and exclusion criteria. The inclusion criteria for this systematic review and meta-analysis were articles focusing on the prevalence, risk factors, and antimicrobial resistance of poultry salmonellosis, published in English between 2017 and 2024. Eligible studies included those with a cross-sectional design, full-text articles, poultry populations under any management system, and studies on prevalence conducted using ISO 6579 for isolation and culturing. Studies on antimicrobial resistance used the Kirby-Bauer disk diffusion method (12), which was also an additional inclusion criterion for this study. Furthermore, studies on systematic reviews, meta-analyses, review articles, studies lacking complete information, articles from outside Ethiopia, samples from food origin (raw eggs, milk, and meat produced for consumption), and studies that did not meet the inclusion criteria were excluded from this study.

### 2.4 Study selection and data extraction

**Figure 1:**
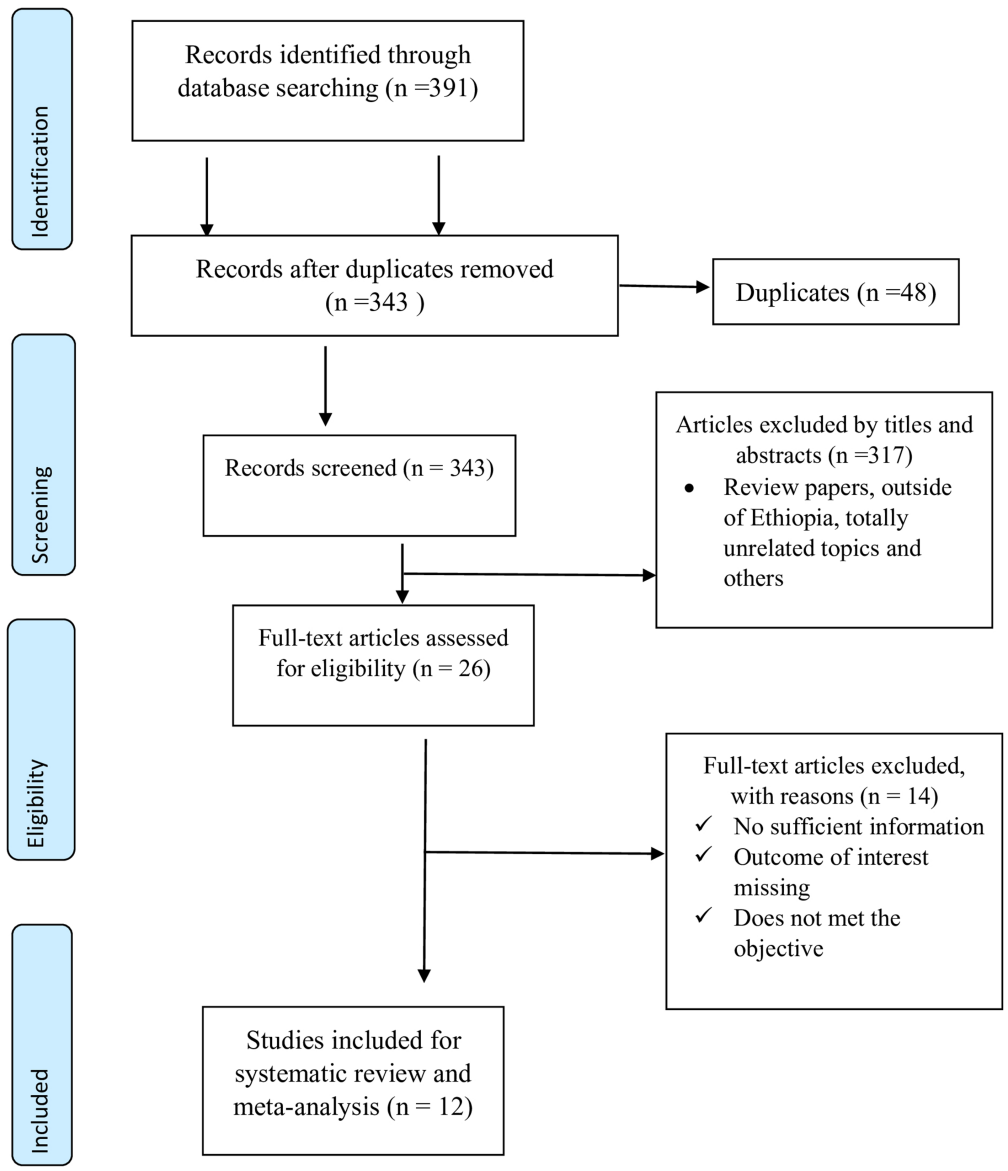
PRISMA flow chart for selection of studies on prevalence, risk factors and antimicrobial resistance of poultry salmonellosis in Ethiopia.

Articles identified from various electronic databases were exported to Zotero 7.0.3. After identifying and removing duplicates, the titles and abstracts were reviewed and cross-checked against the predetermined inclusion criteria. The full texts of the selected articles were thoroughly evaluated for compliance with the inclusion criteria. The articles were screened independently by EB and ATG using the predetermined inclusion criteria. The data were extracted and evaluated independently by EB and ATG. A third researcher, named HD, independently resolved disagreements between the two researchers. Data retrieved from each article included the first author, publication year, geographical location (region and zone), sample size or total number of samples collected, sample type, sampling technique, number of *Salmonella*-positive chickens, diagnostic techniques for antimicrobial-resistant isolates, and the number of antimicrobial-resistant isolates against the selected antimicrobial agents. Studies that did not meet the inclusion criteria were excluded, as illustrated in the PRISMA flow charts (Figure 1).

### 2.5 Quality assessment of the selected studies

Using AXIS critical appraisal tool for quality assessment checklist (13), EB evaluated the quality of the selected articles. The checklist contained 20 elements aligned with key sections of academic articles, including the title, abstract, introduction, methods, results, and discussion. It addressed criteria such as the study’s objective, methodological components (e.g., sample size, demographics, study design, bias mitigation, and statistical methods), findings, and limitations (Supplementary File 2).

### 2.6 Data management and statistical analysis

Relevant data were extracted from the studies included in this systematic review and meta-analysis using Microsoft Excel. The pooled prevalence estimates, antimicrobial resistance, and subgroup analyses were conducted using R software version 4.4.1 (14).

#### 2.6.1 Pooled prevalence estimate

Individual study provided a point estimate of the apparent prevalence of salmonellosis and resistance of antimicrobials. The pooled prevalence (effect size) was determined by dividing the number of positive samples by the entire sample size. A 95% confidence interval for the point prevalence was also generated, as was the standard error, using a specified formula.

$\mathrm{SE}=\sqrt{\frac{1}{n*P*(1-P)}}$ , Where SE = standard error, n = sample size, and p = study-level prevalence estimate.

#### 2.6.2 Assessment of heterogeneity among studies

DerSimonian and Laird (15) technique was used to run a random-effects meta-analysis model using logit-transformed prevalence data. The random-effects model’s inverse variance was used to quantify heterogeneity between and within studies (16, 17).

Heterogeneity between studies was evaluated through the Cochran’s Q test, *I*^2^, and τ^2^.

$Q=\sum (w*{\mathrm{ES}}^{2})-\frac{{\left[\sum (w*\mathrm{ES})\right]}^{2}}{\sum w}$ , Where w = weight of the individual study and ES = effect size/logit prevalence.

The inverse variance index (*I*^2^) indicates how much difference between studies is due to real heterogeneity rather than chance. I^2^ value of 0% implies no observable heterogeneity, while values of 25, 50, and 75% indicate low, moderate, and high levels of heterogeneity, respectively. τ^2^ showed an estimate of the true variance effect size between studies (18).

$I^{2}=\frac{Q-\mathrm{df}}{Q}*100$ , df = degree of freedom (n-1), n = number of studies, Q = Cochran Q statistics.

Individual study weights ( w ) were calculated as the inverse of their variance ( $w=\frac{1}{SE^{2}}$ ) to estimate the pooled prevalence of salmonellosis and resistance of antimicrobials in the chicken population. A random-effects model meta-analysis with a 95% confidence interval was performed (49).

A forest plot was used to represent the overall prevalence estimate for individual studies. The horizontal lines in the plot represent confidence intervals, while the colored boxes show the results for the point estimates of an individual study.

#### 2.6.3 Subgroup meta-analysis

A subgroup analysis of poultry salmonellosis prevalence was conducted based on various factors, including age group (<6 months, 6–12 months, and >12 months), study area (Southern Ethiopia, Central Ethiopia, and Western Ethiopia), breed (Bovans Brown, White Leghorn, Sasso, Cobb 500, and local breeds), housing system (deep litter and cage), purpose of production (meat or egg production), and sample type (cloacal swab, bedding, personnel hand swab, feed, water, fecal droppings, cecal samples, and floor swab). These analyses aimed to identify potential sources of heterogeneity among studies. A random-effects model was used for the analysis at a 95% confidence interval. The inverse variance index (*I*^2^), true variance (*τ*^2^) and chi-square across the group was estimated to assess the significance of heterogeneity among studies, with a significance level set at *p* < 0.05.

#### 2.6.4 Assessment of the presence of publication bias

The presence of potential publication bias in quantitative synthesis was assessed qualitatively (effect size with standard error) using funnel plots and statistically using Egger’s and Begg’s tests (19).

## 3 Results

### 3.1 Search results

A total of 391 potentially relevant research studies were identified from a wide range of sources, including Medline/PubMed, ScienceDirect/Scopus, Google Scholar, Web of Science, and Science Pub. After removing 48 duplicated articles using Zotero and manual tracing, 343 records remained. These were screened based on their titles and abstracts, resulting in the exclusion of 317 articles. The remaining 26 articles, selected through title screening, were further evaluated by reviewing their full text against the eligibility criteria. As a result, 14 articles were excluded due to insufficient information, missing outcomes of interest, or failure to meet the study’s objectives. Ultimately, 12 articles met the eligibility criteria and were included in the systematic review and meta-analysis (Figure 1).

### 3.2 Study characteristics

The 12 published articles that were considered eligible for assessing the pooled prevalence of salmonellosis in poultry are shown in Table 1. These studies were published between 2017 and 2024, and all were cross-sectional in design. The majority of the studies were conducted in central, southern, and western Ethiopia, with no studies on salmonellosis prevalence in poultry available from northern and eastern Ethiopia. The studies involved different poultry breeds, including 3 articles on Bovans Brown, 2 articles on White Leghorn, 3 articles on Sasso, 2 articles on Cobb500, and 2 articles on local breeds. The highest positivity rate reported across all the studies was 24.3% (131/539), while the lowest was 2.9% (24/836). Most studies used microbiological culture procedures (ISO 6579, 1998–2002), with a few also incorporating PCR and serum agglutination methods (Table 1).

**Table 1:** Description summary of the selected 12 studies describing the prevalence, risk factors and antimicrobial resistance of poultry salmonellosis in Ethiopia.

ID	Author	Study area	Breed	Diagnostic technique	Sample size	Positive sample	Prevalence (%)
1.	Abdi et al. (9)	Southern Ethiopia	Bovans and White Leghorn	ISO2002(C-B)	270	45	16.7
2.	Mohammed and Dubie (47)	Central Ethiopia	Bovans and White Leghorn	ISO2002(C-B)	200	23	11.5
3.	Abda et al. (34)	Southern Ethiopia	White leghorn and Sasso	ISO2002(C-B)	302	28	9.27
4.	Eguale (35)	Central Ethiopia	Exotic	ISO2002(C-B) Serum agglutination	549	26	4.7
5.	Asfaw et al. (36)	Central Ethiopia	Exotic and local	ISO2002(C-B)	384	56	14.6
6.	Dagnew et al. (37)	Central Ethiopia	Exotic and local	ISO2002(C-B)	836	24	2.9
7.	Akalu et al. (38)	Central Ethiopia	Exotic and local	ISO2002(C-B)	471	58	12.3
8.	Belachew et al. (39)	Central Ethiopia	Ross 308, Hubbard classic and Cobb 500	ISO2002(C-B)	539	131	24.3
9.	Asmamaw et al. (48)	Western Ethiopia	local and exotic breed	ISO1998(C-B)	384	89	23.2
10.	Waktole et al. (40)	Central Ethiopia	Bovans, Cobb-500, and Sasso	ISO2002(C-B) and PCR	1,515	218	14.4
11.	Sarba et al. (41)	Central Ethiopia	local and hybrid breeds	ISO2002(C-B) and slide agglutination	946	113	11.9
12.	Abayneh et al. (42)	Southern Ethiopia	Sasso and Bovans Brown	ISO2002(C-B)	390	21	5.4

### 3.3 Quality assessment result of selected articles

The quality score of the included articles ranged from 80 to 95% based on the AXIS critical appraisal checklist for studies reporting prevalence data. Among the 12 articles included in this systematic review and meta-analysis, only one article met 19 out of the 20 criteria. Of the remaining 11 articles, nine met 18 out of 20 criteria, while the last two fulfilled 16 out of 20 criteria. None of the studies met all 20 criteria.

### 3.4 Quantitative synthesis of the selected studies

**Figure 2:**
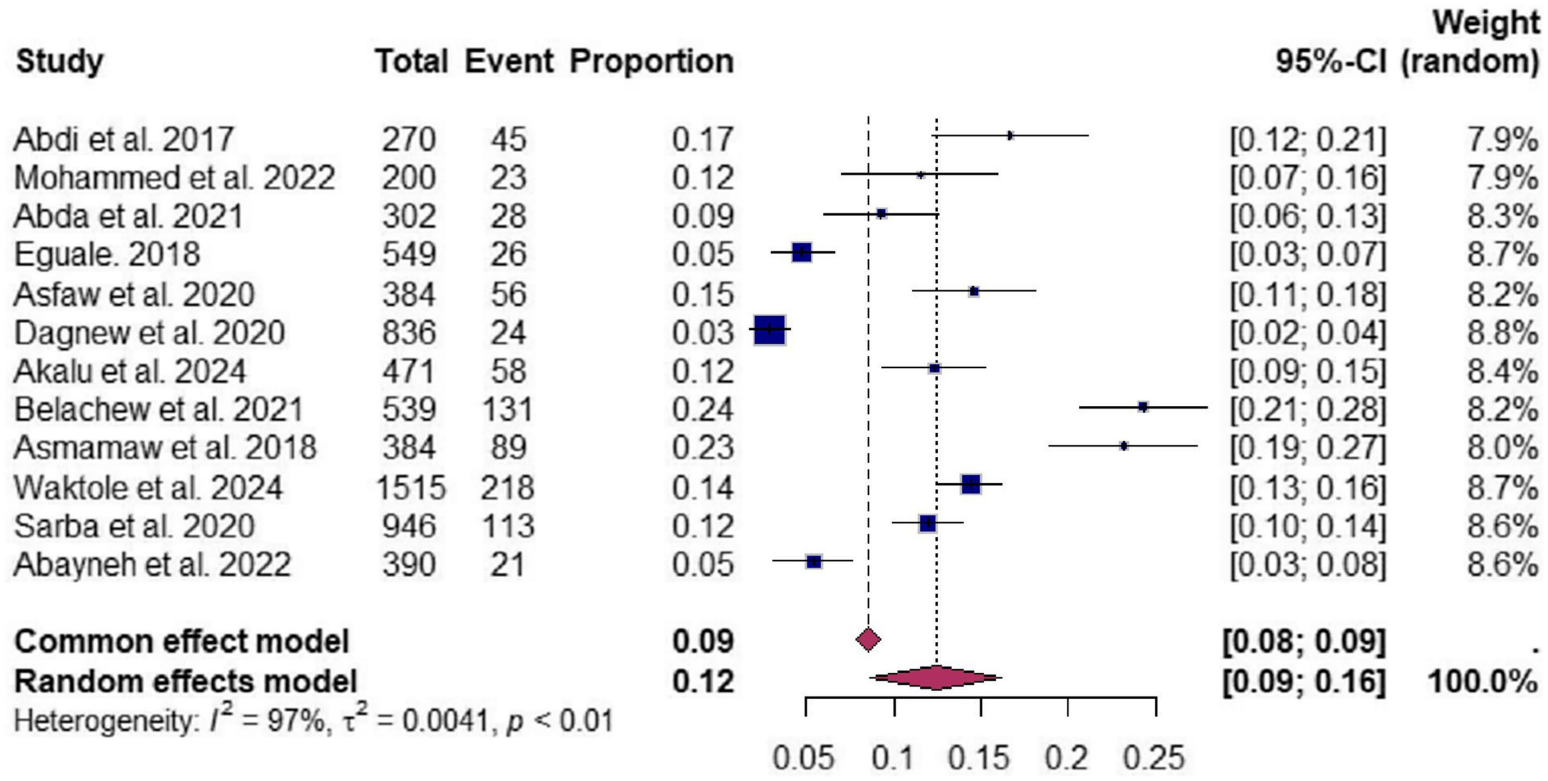
Forest plot depicting the pooled prevalence of poultry salmonella in Ethiopia including 12 studies selected for meta-analysis.

The overall results of the meta-analysis are presented in Figure 2. In this systematic review and meta-analysis, the pooled prevalence of *Salmonella* among poultry in Ethiopia was 12.46% (95% CI: 8.44–16.48). Furthermore, the reported pooled prevalence demonstrated significant heterogeneity among the studies (τ^2^ = 0.0041, H^2^ = 30.19, I^2^ = 96.6%, df = 11, *p* ≤ 0.001).

### 3.5 Subgroup meta-analysis

The results of the subgroup analysis based on study location are presented in Table 2. The highest pooled prevalence of *Salmonella* was observed in Western Ethiopia (23.18, 95% CI: 8.96–37.39), followed by Central Ethiopia (11.98, 95% CI: 7.08–16.88) and Southern Ethiopia (10.3, 95% CI: 2.22–18.38) (Supplementary Figure 1).

**Table 2:** Subgroup analysis for comparison of the pooled prevalence of poultry salmonellosis based on study area.

Study location	Prevalence (95% CI)	I^2^	Tau	*P*-value
Southern Ethiopia	10.30% (95% CI: 2.22, 18.38)	90.2%	0.0693	<0.01
Central Ethiopia	11.98% (95% CI: 7.08, 16.88)	97.3%	0.0693	<0.01
Western Ethiopia	23.18% (95% CI: 8.96, 37.39)	-	0.0693	<0.01
Overall	12.46% (95% CI: 8.44, 16.48)	96.6%	0.0693	<0.01

The results showed statistically significant differences in *Salmonella* prevalence across different age groups (Table 3). The highest pooled prevalence was observed in chickens younger than 6 months (14.45, 95% CI: 8.92–19.99), while the lowest was observed in chickens older than 12 months (6.20, 95% CI: 0–15.9) (Supplementary Figure 2).

**Table 3:** Subgroup analysis for comparison of the pooled prevalence of poultry salmonellosis based on age group.

Age group	Number of studies	Prevalence (95% CI)	I^2^	tau	*P*-value
<6 months	8	14.45% (95% CI: 8.92, 19.99)	93.5%	0.0708	<0.01
6–12 months	2	10.03% (95%CI: 0.0, 20.26)	90.1%	0.0708	<0.01
>12 months	3	6.20% (95% CI: 0, 15.9)	56.5%	0.0708	0.1

The subgroup analysis based on breed showed that the pooled prevalence of *Salmonella* was 17.48% (95% CI: 0.00–38.02%) in White Leghorn, 17.88% (95% CI: 1.06–34.69%) in Sasso, 22.21% (95% CI: 5.54–38.88%) in Bovans Brown, 45.26% (95% CI: 23.44–67.08%) in Cobb 500, and 39.78% (95% CI: 19.50–60.06%) in local breeds. The I^2^ values for the logit event estimates were 94.9% for Bovans Brown, 0.0% for White Leghorn, 94.3% for Sasso, 92.6% for Cobb 500, and 98.8% for local breeds (Table 4). The heterogeneity variance was τ^2^ = 0.0201, and the average deviation from the mean effect size was approximately 0.1419 (*p* < 0.01) (Supplementary Figure 3).

**Table 4:** Subgroup analysis for comparison of the pooled prevalence of poultry salmonellosis based on breed.

Breed	Number of studies	Prevalence (95% CI)	I^2^	tau	*P*-value
Bovans brown	3	22.21% (95% CI: 5.54, 38.88)	94.9%	0.1419	<0.01
White Leghorn	2	17.48% (95% CI: 0.00, 38.02)	0.0%	0.1419	0.89
Sasso	3	17.88% (95% CI: 1.06, 34.69)	94.3%	0.1419	<0.01
Cobb 500	2	45.26% (95% CI: 23.44, 67.08)	92.6%	0.1419	<0.01
Local breeds	2	39.78% (95% CI: 19.50, 60.06)	98.8%	0.1419	<0.01

The subgroup analysis based on the housing system is presented in Table 5. Only seven out of the 12 studies reported the housing system of the animals. The remaining five studies did not provide any information regarding the housing conditions. The pooled prevalence of *Salmonella* was 13.45% (95% CI: 5.33–21.56%) in the deep litter system and 12.90% (95% CI: 3.28–22.52%) in the cage system. The I^2^ values for the logit event estimates were 93.5% for the deep litter system and 92.3% for the cage system. The true heterogeneity variance was τ^2^ = 0.0064, and the average deviation from the mean effect size was approximately 0.0798 (*p* < 0.01) (Supplementary Figure 4).

**Table 5:** Subgroup analysis for comparison of the pooled prevalence of poultry salmonellosis based on housing.

Housing	Number of studies	Prevalence (95% CI)	I^2^	tau	*P*-value
Deep litter	4	13.45% (95% CI: 5.33, 21.56)	93.5%	0.0798	<0.01
Cage system	3	12.90% (95% CI: 3.28, 22.52)	93.3%	0.0798	<0.01

The results of the subgroup meta-analysis based on the purpose of production indicate that the pooled prevalence of *Salmonella* was 28.23% (95% CI: 19.97–36.49%) in broilers and 12.77% (95% CI: 5.00–20.54%) in layers. The I^2^ values for the logit event estimates were 98.0% for broilers and 96.8% for layers (Table 6). The heterogeneity variance was τ^2^ = 0.0070, and the average deviation from the mean effect size was approximately 0.0835 (*p* < 0.01) (shown in Supplementary Figure 5).

**Table 6:** Subgroup analysis for comparison of the pooled prevalence of poultry salmonellosis based on purpose.

Purpose	Number of studies	Prevalence (95% CI)	I^2^	tau	*P*-value
Broiler	5	28.23% (95% CI: 19.97, 36.49)	98.0	0.0835	<0.01
Layer	5	12.77% (95% CI: 5.00, 20.54)	96.8	0.0835	<0.01

A subgroup meta-analysis was conducted to examine the pooled prevalence of salmonellosis across different sample types collected from chickens. The results of the random-effects meta-regression model showed the following pooled prevalence: 12.90% (95% CI: 7.42–18.38%) in cloacal swabs, 18.54% (95% CI: 6.74–30.34%) in beddings, 18.45% (95% CI: 0.43–36.48%) in personnel hand swabs, 7.89% (95% CI: 0.18–15.59%) in feed, 11.98% (95% CI: 1.14–22.82%) in water, 8.69% (95% CI: 1.76–15.62%) in fecal droppings, 17.48% (95% CI: 6.06–28.90%) in cecal samples, and 5.21% (95% CI: 0.00–16.48%) in floor swabs. The I^2^ values for the logit event estimates in cloacal swabs, beddings, personnel hand swabs, feed, water, fecal droppings, cecal samples, and floor swabs were 98.0, 0.0, 93.1, 87.9, 72.7, 84.7, 51.8, and 0.0%, respectively (Table 7). The true variance was τ^2^ = 0.0059, and the average deviation from the mean effect size was approximately 0.0766 (*p* < 0.01) (as observed in Supplementary Figure 6).

**Table 7:** Subgroup analysis for comparison of the pooled prevalence of poultry salmonellosis based on sample.

Sample type	Number of studies	Prevalence (95% CI)	I^2^	Tau	*P*
Cloacal swab	8	12.90% (95% CI: 7.42, 18.38%)	98%	0.0766	<0.01
Bedding	3	18.54% (95% CI: 6.74, 30.34%)	0%	0.0766	0.95
Personnel hand swab	2	18.45% (95% CI: 0.43, 36.48%)	93.1%	0.0766	<0.01
Feed	5	7.89% (95% CI: 0.18, 15.59%)	87.9%	0.0766	<0.01
Water	3	11.98% (95% CI: 1.14, 22.82%)	72.7%	0.0766	0.03
Fecal droppings	5	8.69% (95% CI: 1.76, 15.62%)	84.7%	0.0766	<0.01
Caecal sample	2	17.48% (95% CI: 6.06, 28.90%)	51.8%	0.0766	0.15
Floor swab	2	5.21% (95% CI: 0.00, 16.48%)	0%	0.0766	0.33

### 3.6 Pooled estimate of antimicrobial resistance in poultry

The results showed that *Salmonella* strains exhibited varying resistance profiles against selected antimicrobials (Table 8). The meta-analysis revealed a high pooled resistance to tetracycline at 75% (95% CI: 70–79). In contrast, the lowest pooled resistance was observed for cefotaxime at 3% (95% CI: 0–7) (Supplementary Figure 7).

**Table 8:** Antimicrobial resistance patterns of salmonella isolated from poultry in Ethiopia.

Antimicrobials	Number of studies	Number of isolates	Number of resistant isolates	Pooled prevalence (95%CI)
AMP	8	321	179	56% (95%CI 50.61)
AMX	7	299	162	54%(95%CI 49.60)
NIT	3	90	57	63% (95%CI 53.73)
TET	8	337	252	75% (95%CI 70.79)
S	10	413	234	57% (95%CI52.61)
CHL	10	406	174	43% (95%CI 38.48)
AZM	1	25	3	12% (95%CI 0.25)
GEN	8	331	21	6% (95%CI 4.9)
CTX	3	92	3	3% (95%CI 0.7)
NAL	10	399	208	52% (95%CI 47.57)
KAN	8	321	189	59% (95%CI 53.64)
ERT	2	124	33	27% (95%CI 19.34)
CAZ	2	64	5	8% (95%CI 1. 14)
SXT	10	392	173	44% (95%CI 39.49)
OXY	5	154	98	64% (95%CI 56.71)
NEO	3	79	10	13% (95%CI 5.20)
SUL	3	51	97	53% (95%CI 43.63)
CIP	7	232	30	13% (95%CI 9.17)

AMP, ampicillin; AMX, amoxicillin; AZM, azithromycin; CTX, Cefotaxime; CAZ, Ceftazidime; CHL, chloramphenicol; ERT, erythromycin; GEN, gentamicin; CIP, ciprofloxacin; KAN, kanamycin; NAL, nalidixic acid; NEO, Neomycin; NIT, Nitrofurantoin; S, streptomycin; SUL, Sulfisoxazole; SXT, sulfamethoxazole trimethoprim; TET, tetracycline; OXY, Oxytetracycline.

### 3.7 Publication bias assessment

**Figure 3:**
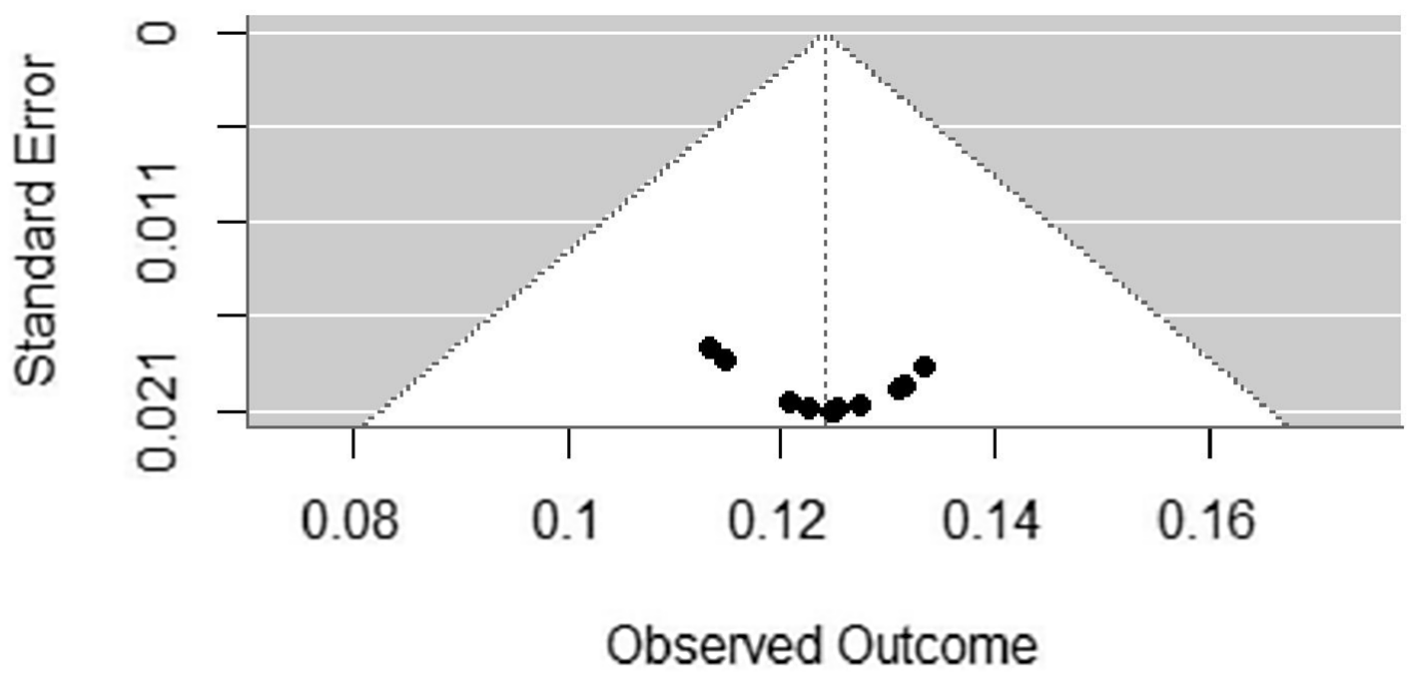
Funnel plot depicting publication bias of studies reporting the pooled prevalence of poultry in Ethiopia including 12 studies selected for meta-analysis.

Publication bias and small-study effects were evaluated using a logit-transformed funnel plot and regression test (Figure 3). The results indicated asymmetry in the slope distribution. The mixed-effects meta-regression test showed no significant publication bias among the included studies (*z* = 0.4178, *p* = 0.6761, *b* = 0.0858, 95% CI: −0.0942, 0.2659).

## 4 Discussion

This systematic review and meta-analysis provide a comprehensive overview and outline the burden of salmonellosis in poultry. A quantitative synthesis of 12 studies, comprising a total of 6,786 samples and 832 *Salmonella* isolates, estimated the pooled prevalence of salmonellosis among poultry in Ethiopia to be 12.46%. For instance, a systematic review conducted in India estimated an overall prevalence of 23% (20) in poultry farms across different regions and, thus, informs that the prevalence in Ethiopia is somewhat comparable to study from India. This may reflect similar agricultural practices. In contrast, a meta-analysis in the United States reported the pooled prevalence at about 6.57% (21). On one hand, this may underline the effect of strict biosecurity measures and regulatory frameworks in controlling *Salmonella* infections in poultry, at least compared to higher rates in developing countries like Ethiopia. It should be underlined that the maximum prevalence of salmonellosis was observed in Cameroon and constituted 93.3% in 2019 (22). This highlights the ongoing challenge of controlling *Salmonella* in certain regions of Africa. Similarly, a study conducted in South Africa showed a prevalence proportion of about 12.1% (23), indicating that while South Africa also faces challenges with salmonellosis, the prevalence is comparable to that in Ethiopia.

A subgroup meta-analysis based on the study area reported that the highest pooled prevalence of salmonellosis was found in western Ethiopia. This result is higher than the prevalence reported by Taddese et al. (50), which was 2.65%. This discrepancy may be due to the limited study conducted in western Ethiopia. Hence, regional variation is still not well understood from this meta-analysis, highlighting the need for further investigation into local practices and environmental conditions that may have contributed to these findings in western Ethiopia. However, the pooled prevalence of salmonellosis in southern Ethiopia was reported to be 10.30%. This result is higher than that of a study conducted in Hawassa, southern Ethiopia, which found a much lower prevalence of 0.8% in apparently healthy chickens, although it reported an isolation rate of 16.1% from sick or dead chickens (24). This deviation might be due to differences in the methodology used during sample collection or the individual health status of the birds tested. In contrast, central Ethiopia exhibited a prevalence of 11.98%. These high prevalence’s could be due to higher poultry densities and different farming practices. This finding can be compared to the study conducted by Kebede and Duga (51), which reported a prevalence of 16.5%. The deviation might be attributed to variations in poultry densities and farming practices in areas around Addis Ababa.

The subgroup meta-analysis based on age reported that the highest pooled prevalence of salmonellosis was found in chicks younger than 6 months (14.45%). In contrast, chickens older than 12 months had the lowest prevalence (6.20%). This result aligns with the age-based prevalence of salmonellosis reported in other meta-analyses from Europe. This shows that younger poultry are more affected by salmonellosis than adults. In the EU, a study showed that young children, 0–4-year-olds, had the highest incidence rates of salmonellosis as compared to the other age groups (43). This variation in prevalence across age groups suggests that older birds may have acquired immunity or benefit from better management practices, while younger birds are at higher risk due to incomplete immune development and greater susceptibility to infections (25).

Subgroup meta-analysis by breed showed the highest pooled prevalence of salmonellosis in Cobb 500 (45.26%) and local breeds (39.78%). In contrast, White Leghorn and Sasso had relatively lower prevalence proportions of 17.48 and 17.88%, respectively. Meanwhile, Bovans Brown had a higher prevalence of 22.21%. The variation in prevalence across breeds, particularly in Cobb 500 and local breeds, may be due to Cobb 500’s popularity, rapid growth rate, and high meat production capacity factors that make it more vulnerable to intensive production methods and poor biosecurity measures (30). Similarly, while local breeds are generally more resistant to local conditions, they appear to have a higher risk of salmonellosis. This suggests that traditional management practices may not be sufficient to control the disease (26). The moderately high prevalence in Bovans Brown may be due to specific environmental or management factors, which should be investigated to improve biosecurity measures for this breed. In contrast, the lower infection rates in White Leghorn and Sasso breeds suggest the presence of innate resistance factors or more effective management practices in preventing contamination (27).

Subgroup meta-analysis based on housing systems reported the highest pooled prevalence of salmonellosis in deep litter systems (13.45%) and cage systems (12.90%), with a rate difference of 0.55% between the two. This result closely aligns with findings from a study conducted in Chile (28), which reported *Salmonella* prevalence in deep litter systems to be 1.1% higher than in cage systems. A meta-analysis from the United States reported a prevalence difference of up to 5% between the two production systems (52). The similarity between the Ethiopian data and the Chilean study suggests that environmental and management factors may influence *Salmonella* survival and transmission in both regions. The greater disparity observed in the U.S. may be due to stricter separation and standardization of housing systems, where each system is managed under distinctly different conditions. In countries like Ethiopia, where poultry farming is often small-scale and management practices may overlap across systems, the operational boundaries between deep litter and cage systems can be less pronounced. This blending of practices may reduce the observable impact of housing type on Salmonella prevalence, resulting in a smaller difference between the two. Research conducted in Spain indicated that non-cage layer systems had significantly higher *Salmonella* incidence compared to caged systems, highlighting the importance of housing conditions in determining the extent of contamination (29).

The results of the subgroup meta-analysis based on production purpose show a significant difference in pooled prevalence between broilers (28.23%) and layers (12.77%). This finding aligns with a meta-analysis conducted in the EU, which reported that broiler flocks consistently have a higher *Salmonella* prevalence than layers, reaching up to 30% (53). This variation in prevalence between broilers and layers is due to the effects of intensive farming systems, higher stocking density, and other factors that often lead to a higher incidence of stress-related diseases in broiler operations (30). Additionally, layers tend to have longer production cycles and may benefit from superior biosecurity measures, which often results in lower prevalence levels.

The estimated pooled prevalence of salmonellosis was 12.90% in cloacal swabs, 18.54% in bedding, 18.45% in personnel hand swabs, 7.89% in feed, 11.98% in water, 8.69% in fecal droppings, 17.48% in ceacal samples, and 5.21% in floor swabs. In comparison, a meta-analysis conducted in China reported higher prevalence in litter (25.4%) and feces (16.3%), with a notably lower prevalence in feed (4.8%). This suggests that litter may be a significant reservoir for *Salmonella*, which aligns with the current analysis, highlighting bedding as a high-risk source. On the other hand, the prevalence in feed is comparatively lower, suggesting better management practices in some areas. A systematic review from Africa reported the following pooled prevalence: 14.5% in litter, 17.8% in feed, 11.2% in cloacal swabs, and 15.2% in feces. In comparison to the present analysis, there is major difference in prevalence of *Salmonella* in fecal samples of both studies and minor difference in cloacal swabs. The estimated pooled prevalence of salmonellosis was 12.90% in cloacal swabs, 18.54% in bedding, and 18.45% in personnel hand swabs, indicating that both environmental and human factors play a significant role in the spread of *Salmonella*.

The meta-analysis of *Salmonella* resistance reveals alarming trends in antimicrobial resistance across most antibiotic classes. Pooled resistance rates were 75% for tetracycline, 64% for oxytetracycline, and 63% for nitrofurantoin, highlighting a significant public health concern. These findings align with a systematic review in South Asia, where the overall prevalence of antimicrobial resistance in *Salmonella* was reported at 70%, with notable resistance against nalidixic acid (74.25%) and tetracycline (37.64%) (31). By contrast, the current analysis shows lower resistance rates, such as 44% for sulfamethoxazole-trimethoprim and 3% for cefotaxime, indicating that some antibiotics remain effective while others are highly ineffective. This trend is consistent with a meta-analysis conducted in Southeast Asia, where tetracycline had a high resistance rate of 37.64% (32). Studies conducted in Ethiopia revealed that 100% of the isolates were resistant to ampicillin, while the current analysis shows a resistance rate of 56%. These results may indicate a regional trend of rising resistance to commonly used antibiotics. Although the resistance rate for newer antibiotics like ceftriaxone was only around 16% (33), this suggests that they could still be useful treatment options. However, the very high resistance rates to traditional antibiotics highlight the urgent need for improved antimicrobial stewardship and monitoring practices to combat the emerging wave of resistant *Salmonella* strains.

### 4.1 Limitations of the study

The study has some limitations, including the lack of study from certain regions of the country. Additionally, variations in sample collection methods and testing techniques could influence the comparability of the results. Moreover, prevalence and AMR of chicken-based products are not included in this study for *Salmonella*.

## 5 Conclusion and recommendations

This systematic review and meta-analysis is the first pooled prevalence report on chicken salmonellosis in Ethiopia, highlighting a high prevalence of the disease on poultry farms. Younger birds (<6 months of age) were found to have the highest risk of salmonellosis. Cobb 500 and local breeds exhibited higher pooled prevalence compared to other breeds. Additionally, broilers were more at risk of *Salmonella* contamination than layers. Furthermore, litter samples, personnel hand swabs, and ceacal samples showed higher pooled prevalence than other sample types. The study also found higher antimicrobial resistance against tetracycline, oxytetracycline, and nitrofurantoin in *Salmonella* serotypes. The review identified that age, breed, management practices, and poor hygienic conditions are the important factors contributing to the occurrence of poultry salmonellosis in Ethiopia. To control the high prevalence of salmonellosis, limit access to poultry farms to essential personnel, implement sanitation protocols for visitors and equipment, and quarantine newly purchased birds before introducing them to existing flocks. Additionally, vaccination strategies against *Salmonella* should be implemented, and the use of *Salmonella*-resistant breeds should be encouraged. Furthermore, proper litter and waste management should be ensured, and collaboration with government agencies and NGOs is necessary to improve regulatory frameworks and support for poultry farmers.

## Data Availability

The original contributions presented in the study are included in the article/Supplementary material, further inquiries can be directed to the corresponding author.
