# Supplementary material for: A systematic review and meta-analysis of salmonellosis in poultry farms in Ethiopia: prevalence, risk factors, and antimicrobial resistance
Source: Front Vet Sci. 2025 May 28;12:1538963. doi: 10.3389/fvets.2025.1538963 (PMC12153448; doi:10.3389/fvets.2025.1538963)
Supplement: Supplementary file 2 [file Data_Sheet_2.docx]

**Supplementary file 2**: AXIS Critical appraisal tool for cross-sectional studies

|  | **Question** | **Yes** | **No** | **Don’t know/ Comment** |
| --- | --- | --- | --- | --- |
| [***Introduction***](#_bookmark1) | | | | |
| 1 | [Were the aims/objectives of the study clear?](#_bookmark2) |  |  |  |
| [***Methods***](#_bookmark4) | | | | |
| 2 | [Was the study design appropriate for the stated aim(s)?](#_bookmark5) |  |  |  |
| 3 | [Was the sample size justified?](#_bookmark6) |  |  |  |
| 4 | [Was the target/reference population clearly defined? (Is it clear who the](#_bookmark8) [research was about?)](#_bookmark8) |  |  |  |
| 5 | [Was the sample frame taken from an appropriate population base so that it](#_bookmark9) [closely represented the target/reference population under investigation?](#_bookmark9) |  |  |  |
| 6 | [Was the selection process likely to select subjects/participants that were](#_bookmark11) [representative of the target/reference population under investigation?](#_bookmark11) |  |  |  |
| 7 | [Were measures undertaken to address and categorise non-responders?](#_bookmark12) |  |  |  |
| 8 | [Were the risk factor and outcome variables measured appropriate to the aims](#_bookmark13) [of the study?](#_bookmark13) |  |  |  |
| 9 | [Were the risk factor and outcome variables measured correctly using](#_bookmark13) [instruments/measurements that had been trialled, piloted or published](#_bookmark13) [previously?](#_bookmark13) |  |  |  |
| 10 | [Is it clear what was used to determined statistical significance and/or](#_bookmark14) [precision estimates? (e.g. p-values, confidence intervals)](#_bookmark14) |  |  |  |
| 11 | [Were the methods (including statistical methods) sufficiently described to](#_bookmark16) [enable them to be repeated?](#_bookmark16) |  |  |  |
| [***Results***](#_bookmark17) | | | | |
| 12 | [Were the basic data adequately described?](#_bookmark18) |  |  |  |
| 13 | [Does the response rate raise concerns about non-response bias?](#_bookmark19) |  |  |  |
| 14 | [If appropriate, was information about non-responders described?](#_bookmark19) |  |  |  |
| 15 | [Were the results internally consistent?](#_bookmark20) |  |  |  |
| 16 | [Were the results presented for all the analyses described in the methods?](#_bookmark21) |  |  |  |
| [***Discussion***](#_bookmark22) | | | | |
| 17 | [Were the authors' discussions and conclusions justified by the results?](#_bookmark23) |  |  |  |
| 18 | [Were the limitations of the study discussed?](#_bookmark29) |  |  |  |
| [***Other***](#_bookmark30) | | | | |
| 19 | [Were there any funding sources or conflicts of interest that may affect the](#_bookmark31) [authors’ interpretation of the results?](#_bookmark31) |  |  |  |
| 20 | [Was ethical approval or consent of participants attained?](#_bookmark32) |  |  |  |
