# Supplementary material for: A systematic review and meta-analysis of salmonellosis in poultry farms in Ethiopia: prevalence, risk factors, and antimicrobial resistance
Source: Front Vet Sci. 2025 May 28;12:1538963. doi: 10.3389/fvets.2025.1538963 (PMC12153448; doi:10.3389/fvets.2025.1538963)

Supplementary figure 4: Forest Plot depicting the pooled prevalence of poultry salmonellosis based on housing in Ethiopia

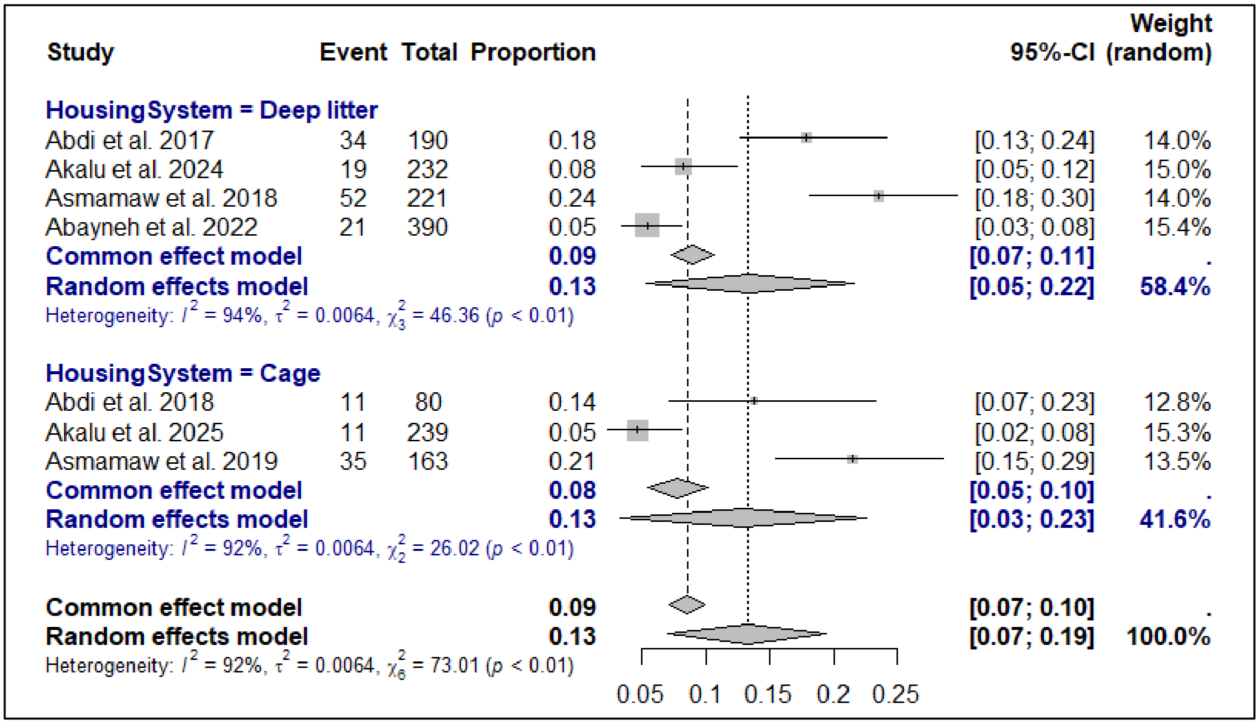

Supplement: Supplementary file 6 [file Image_4.pdf]
