# Supplementary material for: A systematic review and meta-analysis of salmonellosis in poultry farms in Ethiopia: prevalence, risk factors, and antimicrobial resistance
Source: Front Vet Sci. 2025 May 28;12:1538963. doi: 10.3389/fvets.2025.1538963 (PMC12153448; doi:10.3389/fvets.2025.1538963)

Supplementary figure 6: Forest Plot depicting the pooled prevalence of poultry salmonellosis based on sample type in Ethiopia.

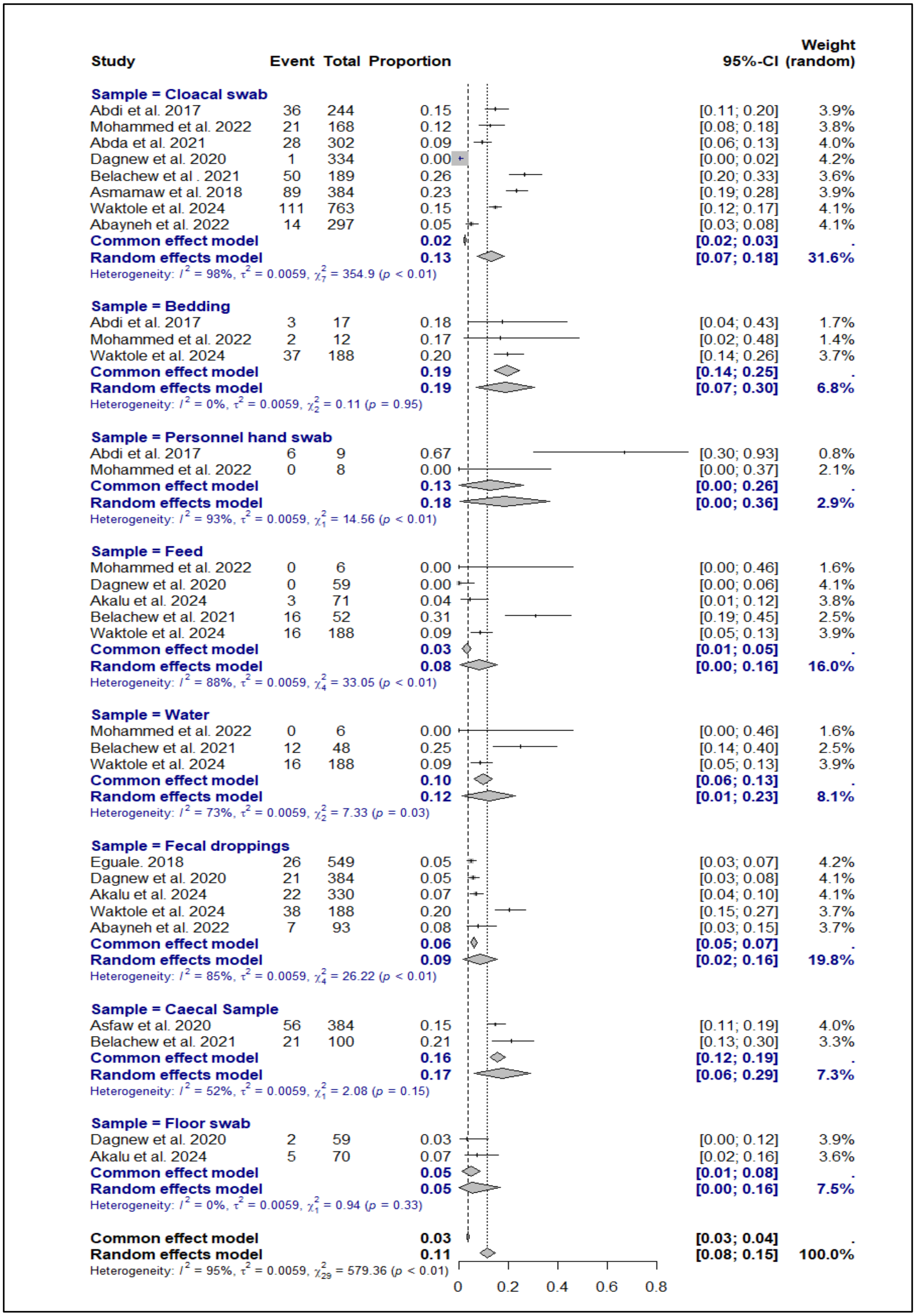

Supplement: Supplementary file 8 [file Image_6.pdf]
